# A Rare Form of Granulomatosis With Polyangiitis With Pyoderma Gangrenosum

**DOI:** 10.7759/cureus.30375

**Published:** 2022-10-17

**Authors:** Tijana Samardzic, Gilana Finogenov, David Podell, Suleyman Felek

**Affiliations:** 1 Internal Medicine, Yale New Haven Hospital, Waterbury, USA; 2 Rheumatology, Waterbury Hospital, Waterbury, USA; 3 Internal Medicine, Yale-Waterbury Internal Medicine Residency Program, Waterbury Hospital, Waterbury, USA

**Keywords:** pyoderma gangrenosum, non-healing ulcer, spinal abscess, anca associated vasculitis, granulomatosis with polyangiitis (gpa)

## Abstract

Granulomatosis with polyangiitis (GPA), formerly known as Wegener's granulomatosis, is rare and poorly understood and can affect a wide range of organ systems and is progressive in nature. Most commonly affecting the respiratory tract and kidneys, it has also been cited to affect the central nervous system (CNS) and skin. Proper and timely diagnosis will warrant appropriate treatment with slowing of disease progression. Our case illustrates a rare form of GPA with CNS and skin involvement that urges a wide differential for proper diagnosis.

## Introduction

Granulomatosis with polyangiitis (GPA) is a rare and poorly understood immune-mediated disorder, affecting small to medium-sized vessels throughout the body, ranging from airways, lungs, kidneys, and various other organ systems [1]. Central nervous system (CNS) and skin involvement have been reported, albeit scarcely. American College of Rheumatology has criteria for diagnosis that do not include positive antineutrophil cytoplasmic antibodies (ANCA), however, in a majority of cases ANCA is positive which assists with diagnosis. We report a rare case of GPA with pyoderma gangrenosum-like ulcers, spinal abscess, sinusitis, and positive ANCA.

## Case presentation

**Figure 1:**
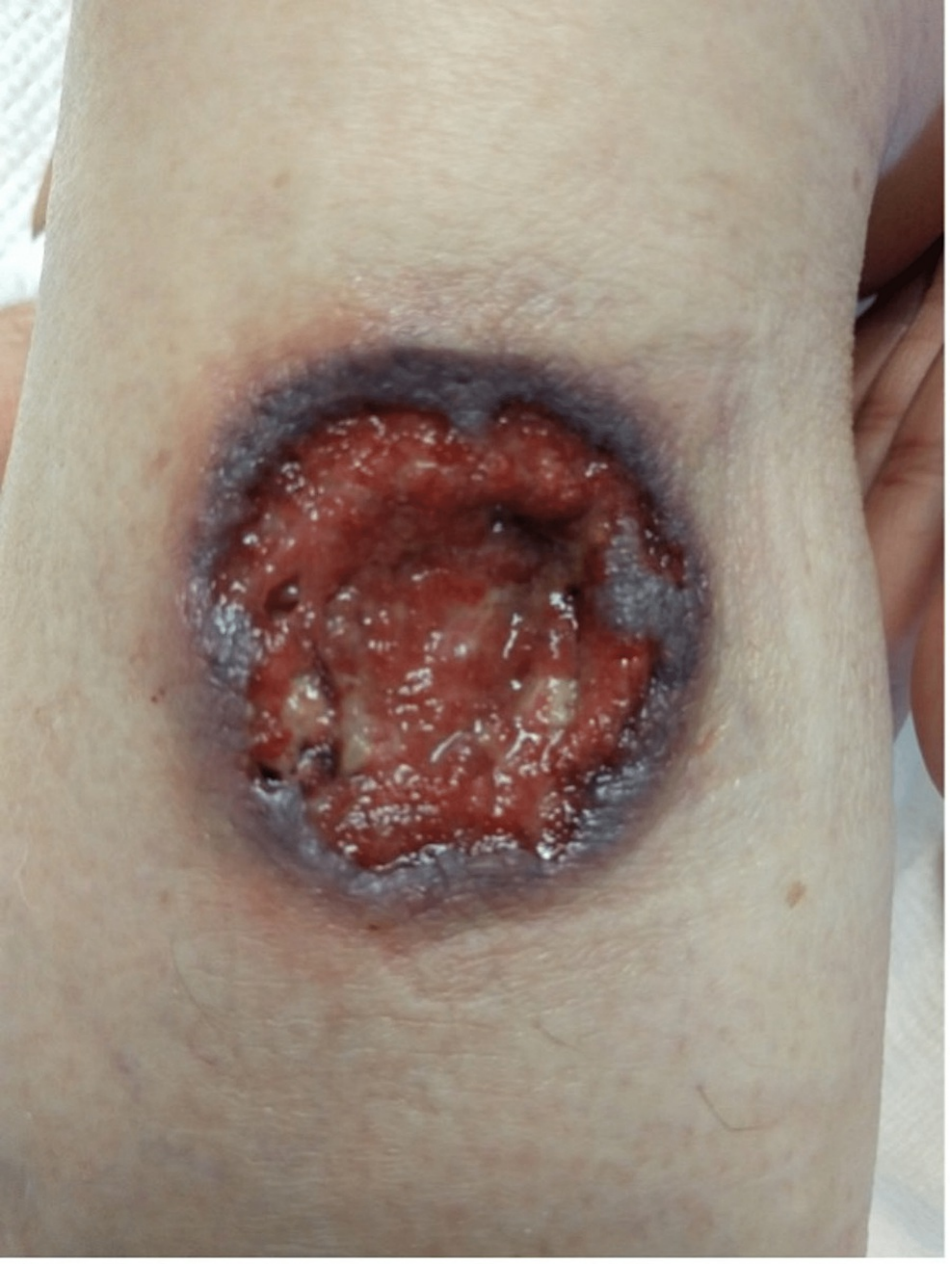
Medial right shin (4x4 cm).

**Figure 2:**
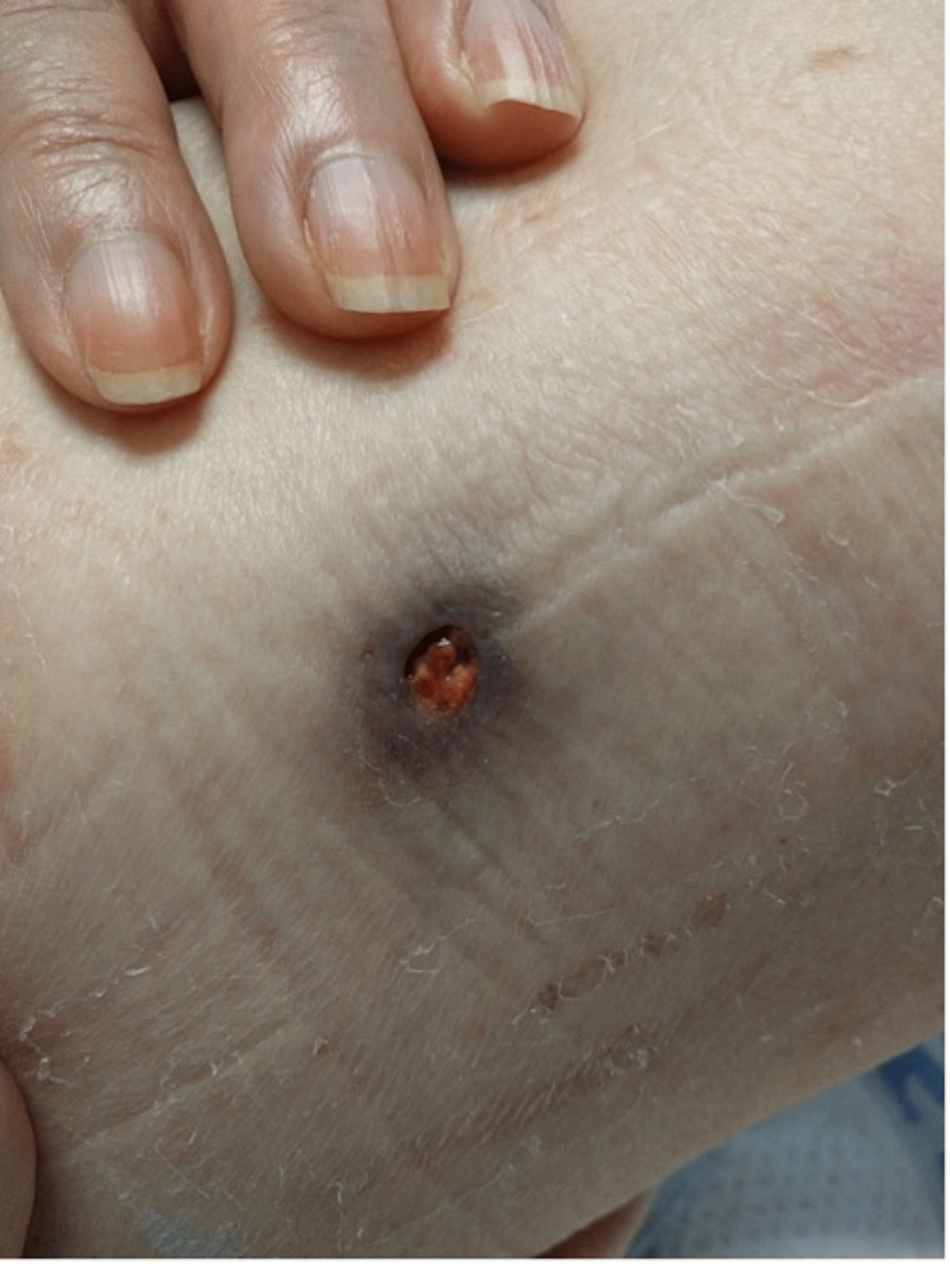
Lateral right thigh (0.5x0.5 cm).

A 63-year-old white, retired saleswoman with a past medical history of recurrent sinusitis presented with relentless, progressive back pain for several days. An initial CT scan was significant for small lytic erosions along T11 and T12 suspicious for osteomyelitis. An MRI demonstrated a phlegmonous prevertebral collection extending from T10 through L1. The patient had concurrently presented with a large non-healing ulcer on her right calf (Figure 1), a smaller non-healing ulcer on her lateral right thigh (Figure 2), as well as a painless subcutaneous nodule on her inner right arm and lower left back (not pictured). The largest ulcer had started as a nodule about six months prior where it sustained repetitive trauma, eventually opening and progressing to become a deeply erythematous 4x4 cm ulcer with a violaceous border and necrotic, granulation tissue in the center.

**Figure 3:**
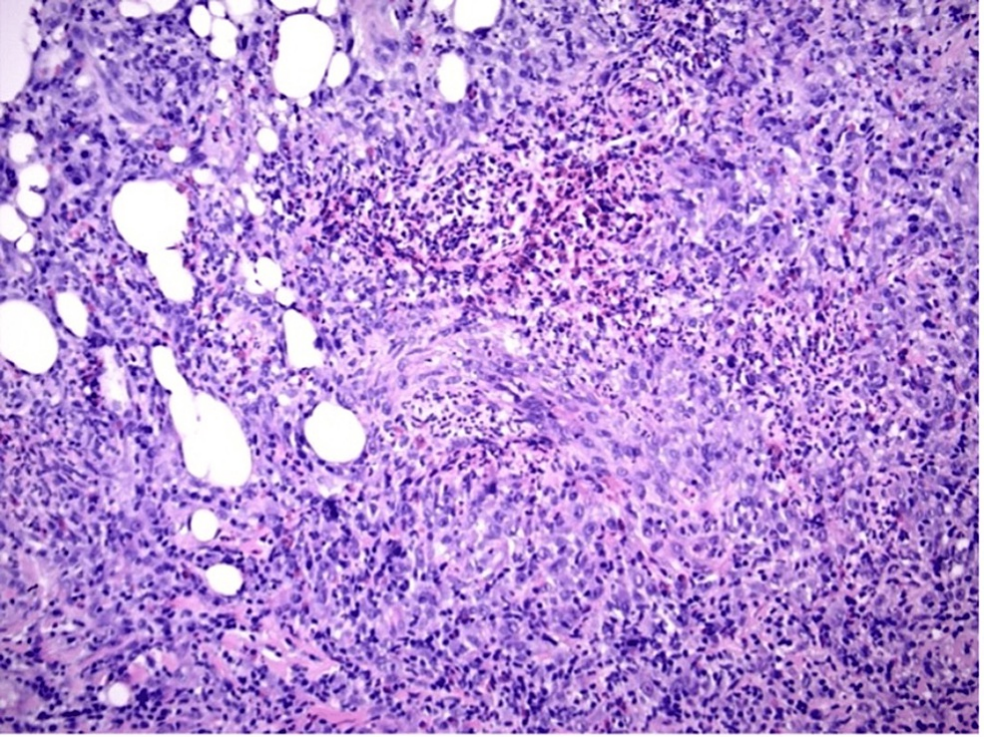
Histological examination of spinal abscess revealing loose granulomatous infiltrates with microabscesses and focal necrosis.

An X-ray of the largest ulcer on her right calf showed no extension to the bone. A deep wound punch biopsy was performed where the pathology had shown microabscesses and focally diffuse neutrophilic infiltrates, histiocytic inflammatory response, as well as scattered multi-nucleated giant cells with granulomatous formation (Figure 3). With concern for osteomyelitis or bacterial seeding, a CT-guided core biopsy was performed on the spinal collection and pathology revealed chronic inflammation, including aggregates of small lymphocytes, plasma cells, and foamy histiocytes, associated with fat necrosis without notes of granulomas. An infectious disease workup of both areas, including mycobacterium, fungal organisms, and bacterial and viral organisms, was negative.

**Figure 4:**
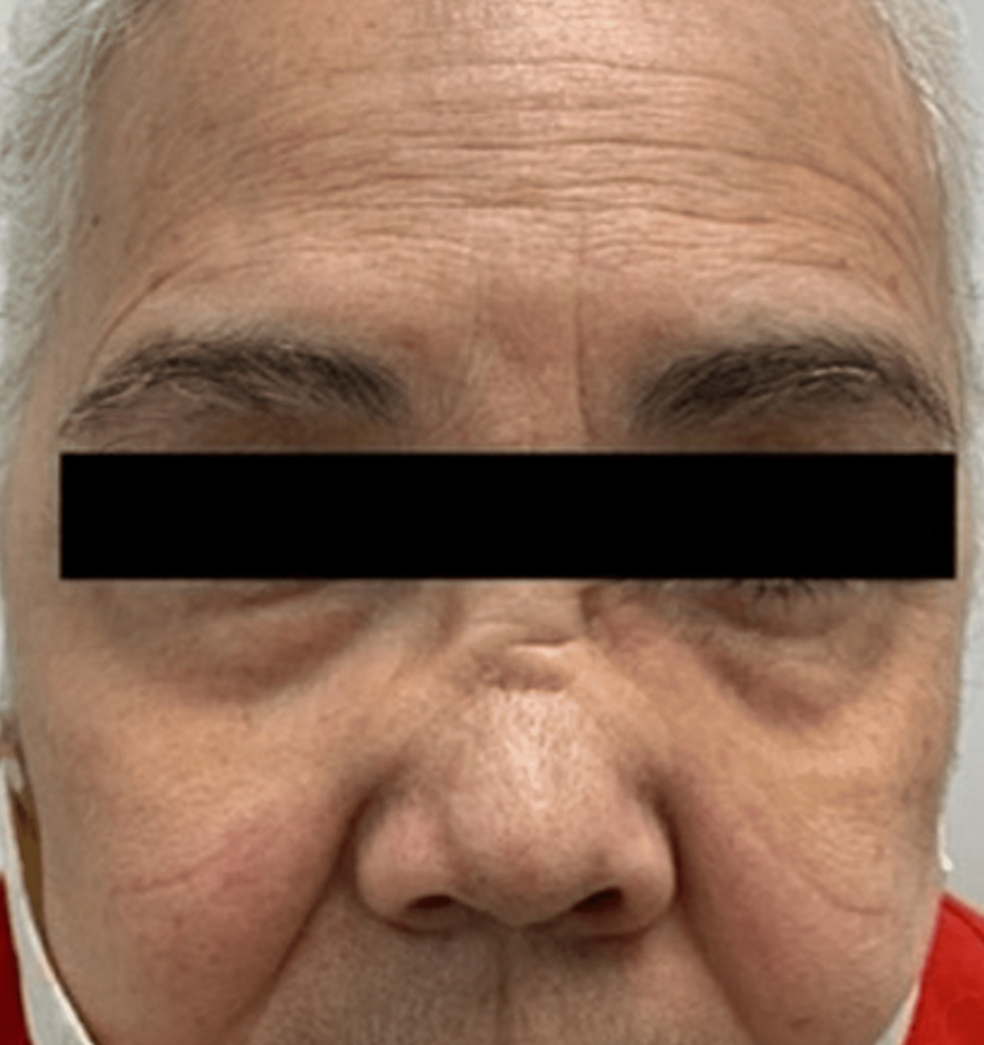
Saddle-nose deformity.

Immunologic studies included an antinuclear antibody (ANA) titer of 1:640 with a homogenous pattern, a Complement-4 (C4) of 50 and C3 of 160 as well as a positive Proteinase-3 Antineutrophil Cytoplasmic Antibodies (PR3-ANCA). Chest X-ray, kidney function, and urinalysis were normal. On further investigation, we obtained sinus pathology reports from a prior sinus surgery early the same year that showed fragments of bone, respiratory mucosa, and extensive chronic inflammation with focal necrosis, with numerous eosinophils. Given her presentation of sinusitis with biopsy reports of inflammation and necrosis, pathology of ulcers and spinal abscess revealing granulomatous change with chronic inflammation, and positive Pr3-ANCA, a diagnosis of Granulomatosis with Polyangiitis with Pyoderma Gangrenosum-like lesions was made. She was managed with prednisone and methotrexate with an initial good response but about two months later, had developed nasal ulceration with the start of progression to saddle nose deformity (Figure 4). She was then switched over to rituximab treatment due to the progressive disease with a good response.

## Discussion

Granulomatosis with polyangiitis (GPA), previously known as Wegener’s granulomatosis, is a disease most commonly affecting small-sized blood vessels throughout the body, usually in the sinuses, lungs, and kidneys [1] and rarely cited in skin and CNS. It is immune-mediated by autoantibodies directed against the neutrophilic granules (PR3-ANCA) thereby causing tissue damage [1-7].

One of two diagnostic criteria, per the American College of Rheumatology (ACR), must include two out of four of the following: (1) nasal/oral inflammation, (2) nodules, fixed infiltrates, or cavities shown in chest X-rays, (3) urinary sediment of red cell casts shown in the urinalysis, and/or (4) granulomatous inflammation shown on biopsy of arteries or perivascular areas. Having two out of four criteria represents an 88.2% sensitivity and a 92% specificity to the disease [3]. Although a PR3-ANCA is not required within the diagnostic criteria, it has >90% specificity in diagnosing GPA [4]. Our patient's initial presentation was not clearly within the diagnostic criteria of GPA and the positive PR3-ANCA persuaded us of its presence and was later confirmed when she developed saddle-nose deformity.

All parts of the body can be affected, however, the most typical presentation includes the upper respiratory tract. It can include recurrence of sinusitis with inflammation that can resemble a severe common cold with persistent rhinorrhea, nasal crusting, nasal congestion or bleeding, as well as ulcerations of mucous membranes [6-8]. Another typical organ system involved is the kidneys, in which patients can develop hypertension, and peripheral edema, which can eventually lead to a more serious condition, glomerulonephritis.

Less common organ systems involved include the skin which can present as papules, subcutaneous nodules, ulcers, petechiae, or purpura and is seen in about 15% of patients [5]. Although skin lesions can be seen, intense ulcerations, like that of pyoderma gangrenosum, are not typical [6].

Even rarer presentations include neurological and cardiac abnormalities, ranging from peripheral neuropathy to hemiplegia and pericarditis, cardiomyopathy, and myocardial infarction. Other generalized systemic symptoms include fevers, malaise, polyarthralgia, loss of appetite, and general fatigue [8-9].

Spinal involvement occurs in about 2% of patients, and although the biopsy in our case seemed inconclusive of vasculitis, over 50% of biopsies related to GPA can be considered nonspecific. The most typical pattern of GPA, however, includes granulomatous formation with necrosis [10] which was seen in our patient's biopsy reports.

As described, there are several rare presentations of this complicated and poorly understood disease and it is important to keep a broad differential when evaluating patients. Our patient had come in for evaluation of back pain that revealed an abscess on imaging, so naturally, our initial concern was for infection. However, on further investigation of her leg ulcerations and her history of chronic sinusitis, we were able to find the proper diagnosis, treatment, and established follow-up of her disease.

## Conclusions

As described, there are several rare presentations of this complicated and poorly understood disease and it is important to keep a broad differential when evaluating patients. Our patient had come in for evaluation of back pain that revealed an abscess on imaging, so naturally, our initial concern was for infection. However, on further investigation of her leg ulcerations, her history of chronic sinusitis, and the labwork that ensued, we were able to tie these findings together to come up with the correct diagnosis and assure timely treatment of the disease. This rare case of GPA with associated skin manifestation of pyoderma gangrenosum-like ulcers was fortunately diagnosed early on with good stability on immunotherapy.
